# Editorial

**Published:** 1874-03

**Authors:** 


### Editorial.

" Here lies the water; good: here stands the man; good: If the man go to this water, and drown himself, it is, will he nill he, he goes; mark you that: but if the water come to him, and drown him, he drowns not himself: argal, he that is not guilty of his own death, shortens not his own life.

" But is this law?

"Ay, marry is't; crowner's-quest law."—*Hamlet, Act V, Scene I.*

Here lies the water; good: here stands the man; good: If the man be carried to the water, and held under the water, once, twice, three times, four times, and be drowned, it is a rupture of the heart, because it was fatty; mark you that.

But is this law?

Ay, marry is't; crowner's-quest law.—*Illinois State Prison Reports.*

Thus has the conjoined wisdom of the State Prison Commissioners, the State Prison Dentist, and the State Prison Coroner's Jury settled the vexed question in æsthetics, and demonstrated that a man may die of a broken heart.

But the cause, think of it! fatty degeneration, and in a convict, too! Truly here is a subject for the investigation of legislative economists. What lives of slothful ease and luxury these convicts must lead. Board and clothing at the expense of the State; dentist's bills reduced to "the cost of the gold," and then to die of fatty degeneration and hydropathy.

A broken heart would certainly be a curiosity in this matter-of-fact age. What has become of the one which once, questionably, graced the poor body of the wretched convict, Henry Williams, and, unquestionably, disgraced the authorities of the Illinois State Prison? It was said to have been submitted to a skillful surgeon, and to an expert pathologist and microscopist of this city. Why have they not been called upon to testify to the facts? They are both as well known for their integrity as for their skill; they will both speak the truth, the whole truth and nothing but the truth, when called upon.

Do the authorities fear this? As the matter stands now, it bears this construction. We have waited patiently for the facts in referto the Williams homicide. We have had, thus far, opinions only; the opinions of the commissioners; of the individual who fills the position of—that is to say, the teeth of the convicts; of the peculiarly disinterested gentlemen who practice hydropathy for the cure of malingering and self-abuse; of that sapient body the coroner's jury; and, also, of volunteers, whose official duties lie in quite another direction. Now, these opinions are all valuable in direct proportion to the public's credulity. Science and Justice demand facts; demand to be assured by competent, and, not only competent, but reliable testimony, what was the condition of the heart of the convict, Henry Williams; was there fatty degeneration, or was it sound? was it ruptured, or was it cut by the scalpel of the careless dissector, running too far through the costal cartilages, or designedly, after the removal of the organ, to make that appear an accidental homicide, which uncharitable people might call a murder? Until these facts are adduced and established, these same uncharitable people will continue to call the act, by which Henry Williams was "cut off even in the blossoms of his sin, unhousel'd, disappointed, unanel'd; no reckoning made, but sent to his account with all his imperfections on his head," murder. H.

***A few Words to Correspondents.***

Please don't write to us, asking that "my name be added to the "long list of subscribers to your valuable journal, and send speci- "men number by mail to my address, when I will remit amount of "subscription." This would necessitate a ride of a mile and a half to our Publishing House, and a loss of time worth more than the annual subscription to the Journal. You can accomplish your object *tuto cito et jucundo* by remitting the amount of the subscription directly to the publishers, who will immediately, on its receipt, forward the JOURNAL to your address.

Please don't send us long speeches on festive occasions, with the request, to "return the manuscript." These belong to the department of "rejected addresses," the return postage upon which would necessitate serious alterations in the Post Master General's estimates.

Send reports of your own cases in practice. Not the rare and remarkable ones only, but those of every-day occurrence; as it is these, and not rare cases, that medical men have to treat. Reports of epidemics possess great value, and should include history, locality, personality, and surrounding circumstances of first cases; course and progress of disease; symptomatology; modes of treatment, both successful and unsuccessful; duration, mortality, etc.

There can be no doubt that practical experiences of physicians, if carefully recorded, would have, long since, furnished sufficient data for the deduction of new principles, and for the decision of many questions *adhuc sub judice;* and it is impossible to say to how great an extent the progress of medical science has been retarded by the neglect to report cases in practice.

In order to do justice to our correspondents, we publish their communications in the order of their reception (with very rare exceptions); and hence are compelled to withold from publication valuable papers for several months; and, in order to do justice to our readers, we have been compelled to reject a number not adapted for publication in our columns.

The JOURNAL is designed to be an index of advances in medical sciences, a mirror of practice, and the mouthpiece of the workers in the profession, through which they and their labors may become known. It cannot become a vehicle for professional advertising.

***Elixirs.***

We have, on several previous occasions, denounced the habit, indulged in by too many medical men, of prescribing for their patients the various fraudulent compounds known to the drug trade as elixirs. No physician has a moral right to prescribe for a patient an agent of whose composition and probable effects he is totally ignorant; and of the composition of these so-called elixirs he is, for the most part, entirely ignorant. It is true, that for many of them formulæ have been published, by which, it is alleged, they are prepared, and thus a cloaking (in the shape of a label) of legitimacy is assumed. It is likewise true, that many of them are not prepared in accordance with these alleged formulæ, and, moreover, could not be, as they suggest pharmaceutical paradoxes.

The difference between these and the old fashioned patent medicines of Ayer, Brandreth, Holloway, *et id genus omne*—which, by the way, they seem to have nearly supplanted—is simply a difference of form, not of principle. The best that can be said in their favor is, that the majority of them are utterly worthless, as far as any therapeutic activity is concerned.

We are very glad to see that the American Pharmaceutical

Association has taken up the consideration of this traffic, with a view to its suppression. The report of its committee, appointed at its last—the twenty-first annual—meeting, has just been received from Prof. Maisch, the permanent Secretary of the Association. The report contains numerous formulæ for the preparation of elixirs, which are proposed for adoption into the Pharmacopœia, and which will have the merit of being definite, and should become officinal. By their adoption, the medical profession will be spared the humiliation of looking to Messrs. A., B., or C., manufacturing druggists, for therapeutical suggestions. The report referred to is republished in detail in the *Pharmacist* for February, 1874. H.

***Law vs. Physic.***

A writer in the *Pharmacist*, in the course of a long article, the gist of which seems to be that he has had too much law in his physic, takes occasion to ridicule the act of the legislature, restricting the sale of poisons. The necessity for such a law is thus demonstrated: In the month of April, 1873, a lady of this city applied at three prominent pharmacies for two ounces of laudanum, the sale of which was refused to her on the ground of its dangerous nature. A fourth was found, whose dispenser, less scrupulous, furnished to a lunatic—unknown to him, of course—the means by which she destroyed her own life within a few hours. The self-satisfaction of the first three, upon learning their own escape from implication in such a tragedy, seemed to be ample compensation for the loss of so small a sale.

The law, moreover, is nothing more than an attempt to enforce upon *all* the observance of that degree of caution which has been habitually exercised by our best pharmacists spontaneously for many years.

Further, the same writer remarks: "In reference to Section 2 of the law forbidding the sale of abortifacients, etc., it need only be stated that 'any person' can buy, throughout the State of Illinois, any amount of 'pills, powders, drugs, or combinations of drugs, designed expressly for the use of females,' etc., etc., etc."

Now, to assert, or even to prove, that there are scoundrels here, as elsewhere, who, for filthy lucre, will infringe or evade a law, is certainly a very poor argument against the justice of the law, or the wisdom of its enactment. Moreover, we are scarcely willing to believe that so many of his own profession are so demoralized and debased, as this writer would seem to imply.

The city of Chicago swarms with thieves: are all laws against theft ridiculous therefore? Because murders are repeatedly perpetrated, are all laws for the protection of human life for that reason absurd?

To the strictly conscientious, the comprehensive formula, "Do as you would be done by," anticipates more specific legislation; he will no more sell poison to a possible suicide or murderer, or an abortifacient to a probable criminal, than he will give to a child a razor for a plaything. To him whose rule of life is the statute-law, and whose motto is "Do, lest you be done," specific legislation is essential, and the rigid infliction of its penalties his only comprehensible argument. H.

***Mortality Statistics.***

Special attention is directed to the Mortality Report at the conclusion of this number. In it we are informed that "the diminution of the mortality is wholly due to the thorough system of vaccination and revaccination employed by the Board of Health, etc., etc. This is undoubtedly true as far as it goes; but the fact is, that the vaccination and revaccination has as much to do with the diminution in the mortality as the excellent quality of Fuseli's colors had to do with the beauty of his painting, for even these would have been inefficient had they not been mixed with "Brains, Sir"! Vaccination and even revaccination without a little administrative ability—brains—in its application, would prove but a very feeble barrier against small-pox, and brains alone will constitute always a strong barrier against any and all epidemics.

We are glad to see the Sanitary Superintendent justifying some of the opinions which we expressed of him at the time of his appointment.

Now let the Council clear out the dead wood amongst his colleagues, and they will learn to their surprise, perhaps, that epidemics, like some other things, can be controlled by those who know how.

